# CircRNA expression profiles in human dental pulp stromal cells undergoing oxidative stress

**DOI:** 10.1186/s12967-019-2078-x

**Published:** 2019-10-01

**Authors:** Jingying Zhang, Dan Li, Dan Wang, Kenny Man, Xuebin Yang

**Affiliations:** 1 The Second Clinical Medical College, Guangdong Medical University, Dongguan, 523808 Guangdong China; 2 College of Life Science and Technology, Dalian University, Dalian, 116622 Liaoning China; 3 College of Medicine, Dalian University, Dalian, 116622 Liaoning China; 4 Biomaterial and Tissue Engineering Group, Division of Oral Biology, School of Dentistry, Wellcome Trust Brenner Building, St. James’s University Hospital, University of Leeds, Leeds, LS9 7TF UK; 5 School of Chemical Engineering, University of Birmingham, Edgbaston, Birmingham, B15 2TT UK

**Keywords:** CircRNA, Oxidative stress, Human Dental Pulp Stromal Cell, Microarray

## Abstract

**Background:**

Oxidative stress has a determinantal effect on human dental pulp stromal cells (hDPSCs), including affecting their longevity and functionality. Circular RNAs (circRNAs) play an essential role in stromal cell behavior; however, the exact mechanism in which circRNAs functions within hDPSCs were undergoing oxidative stress remains unclear. The purpose of this study is to assess the global changes and characteristics of circRNAs in hDPSCs undergoing oxidative stress.

**Methods:**

Using an oxidative stress model of hDPSCs, we applied microarray analysis to examine the circRNAs profiles. We confirmed the changes in circRNAs by quantitative Real-Time PCR (qRT-PCR). Furthermore, bioinformatics tools, including a miRcode map, TargetScan, gene ontology (GO) analysis, Kyoto Encyclopedia of Genes and Genomes (KEGG) pathway analysis, were reconstructed for further assessment. SIRT1 gene and protein expression were tested by qRT-PCR and In Cell-Western analysis.

**Results:**

We revealed 330 upregulated, and 533 downregulated circRNAs undergoing oxidative stress in hDPSCs and confirmed three circRNAs distinct expressions (hsa_circ_0000257, hsa_circ_0087354, and hsa_circ_0001946) in hDPSCs undergoing oxidative stress by qRT-PCR. GO, and KEGG pathway enrichment revealed the differentially expressed circRNAs might participate in p53 and cell cycle signaling networks associated with oxidative stress. SIRT1 gene and protein expression was reduced in the oxidatively stressed cells (OSC) group compared to untreated cells (UC).

**Conclusions:**

The findings of this study has provided new insights into circRNAs and a basis for further studies assessing the potential functions of hsa_circ_0000257, hsa_circ_0087354, and hsa_circ_0001946 in oxidatively stressed hDPSCs.

## Highlights

- Circular RNAs (circRNAs) are markedly altered in oxidatively stressed hDPSCs, including 330 upregulated and 533 downregulated circRNAs, respectively.
- The increased expression levels of hsa_circ_0000257, and decreased expression levels of hsa_circ_0001946 and hsa_circ_0087354 were verified by qRT-PCR.
- The induced hsa_circ_0000257 could interact with the microRNAs, hsa-miR-9-5p, hsa-miR-647, hsa-miR-653-3p, hsa-miR-212-5p, and hsa-miR-27a-5p, enhancing the expression levels of target genes.
- KEGG annotation revealed that p53 signal path might be a critical pathway involved in oxidative stressed hDPSCs, the hsa_circ_0000257/hsa-miR-9-5p/SIRT1/P53 regulatory axis is likely a novel molecular pathway regulating oxidative stress in hDPSCs.

## Background

Human dental pulp stromal cells (hDPSCs) has attracted increasing attention as a mesenchymal stromal cell (MSC) source for regenerative therapy due to their ease and non-invasive acquisition, capacity to self-renew and multipotency [1]. However, hDPSCs are vulnerable to damage by oxidative stress in cell proliferation, and genomic instability and cellular senescence [2]. Although oxidative stress is involved in a wide range of cellular processes, a limited number of studies have examined the effect of oxidative stress in stromal cells, especially in hDPSCs.

Recently, growing evidence has demonstrated circular RNAs (circRNAs), as non-coding RNAs which interacts with microRNAs (miRNAs) [3]. Indeed, circRNAs, also termed miRNA sponges, exert critical functions in gene regulation via a circRNA-miRNA-mRNA pathway, in virtually all mammals [4]. Furthermore, circRNAs are known to participate in oxidative stress [5], which may play a critical role in post-transcriptional gene regulation in oxidative stressed stromal cells. Therefore, circRNAs are becoming crucial biological molecules for understanding the mechanisms of oxidative stress in hDPSCs.

To explore the potential roles of circRNAs in regulating oxidative stress in hDPSCs, we established an oxidative stress model and performed microarray analysis to explore dysregulated circRNAs in oxidative stressed hDPSCs induced by H_2_O_2_ treatment. It will be critical for understanding the regulatory mechanisms of oxidative stress in hDPSCs for regenerative medicine.

## Methods and methods

### Isolation and culture of hDPSCs

Sound third molars were extracted at Affiliated Zhong Shan Hospital of Dalian University and were obtained with patients’ informed consent according to the current study, which had approval from the Research Ethics Committee (No. 2017046). A total of 8 teeth were collected from both male and female patients, with an average age of 24 ± 4 years (mean ± SD). Isolation of hDPSCs was undertaken following the procedure described by Tomlinson et al. [7]. Extracted hDPSCs were cultured in the growth medium of alpha-modified minimum essential medium (α-MEM; 8118353, Gibco), containing 10% fetal bovine serum (FBS; 7981220, NQBB) with 1% penicillin–streptomycin (SV30010, HyClone), at 37 °C in an incubator (Binder, Germany) with 5% CO_2_ and 95% air. When cells reached 80% confluence, they were treated with 0.25% trypsin–EDTA (10525E16, Gibco) and reseeded into multiwell plates for the subsequent experiments.

### H_2_O_2_ treatment

For the induction of oxidative stress, hDPSCs were cultured with freshly prepared H_2_O_2_ in the growth medium. Briefly, 30% H_2_O_2_ was diluted to 1 M stock using sterilized ddH_2_O_2_. Following which, 1 M H_2_O_2_ was further diluted with growth medium at required concentrations and then added to cells  and incubated for 24 h. The cells were washed three times with growth medium to remove residual H_2_O_2_, cultured in fresh growth medium and subjected to subsequent experiments for various durations.

### Oxidative stress model of hDPSCs

CM-H_2_DCFDA (C-6827, Life Technologies) in 10 μL dimethyl sulfoxide (DMSO, CLS3085, Sigma-Aldrich) was further diluted in 5 mL α-MEM and added to the serum-free medium at the concentration of 17.4 μM and incubated at 37 °C in the dark for 30 min. Cells were seeded onto 8-chamber culture slides (Corning, Falcon culture slides) at a density of 5 × 10^4^ cells per cm^2^. After the cells were treated by H_2_O_2_ as described above, the cells were washed three times with PBS and assessed by fluorescence microscopy (AX-10, ZEISS) (Excitation/Emission: 492-495/517-527 nm). Reactive oxygen species (ROS) and SOD activity were detected by Reactive Oxygen Species Assay Kit (Beyotime Biotechnology, China) following the manufacturer’s protocol. The cells were stained by F-actin probe (Alexa Fluor^®^ 568 phalloidin, Invitrogen™) and ProLong^®^ Gold Antifade Mountant with DAPI (P36935, Invitrogen™) following the manufacturer’s protocol.

### RNA extraction

Total RNA extraction from  oxidatively stressed cells (OSC) and untreated cells (UC) was performed with TRIzol (DP405-02, Tiangen) as directed by the manufacturer’s instructions. Following which, RNA quantity and purity were assessed on a NanoDrop spectrophotometer (DNAmaster, dynamica).

### CircRNA microarray analysis

The hDPSCs (OSC and UC groups) were assessed by microarrays to identify differentially expressed circRNAs under oxidative stress. Double-stranded cDNA (ds-cDNA) from 5 μg total RNA was obtained with a SuperScript ds-cDNA synthesis kit (Invitrogen, USA) as instructed by the manufacturer. Following which, ds-cDNA was then labelled with Cy3 as described by the NimbleGen Gene Expression Analysis protocol (NimbleGen Systems, USA), using 1 μg ds-cDNA, 100 pmol of deoxynucleoside triphosphates and 100 U of the Klenow fragment (New England Biolabs, USA). Purification of the labeled ds-cDNA was carried out by ethanol precipitation. Hybridization was performed at 42 °C for 16–20 h using 4 μg of Cy3 labeled ds-cDNA with NimbleGen hybridization buffer/hybridization component A (NimbleGen Systems). Finally, slide scanning was performed on an Axon GenePix 4000B microarray scanner (GenePix 4000B, US Molecular Devices) with the GenePix Pro 6.0 software. The obtained TIFF image files were imported into the NimbleScan software (v2.5) for analyzing the data, which were further assessed with Agilent GeneSpring GX (v12.1). Hierarchical clustering was carried out with R scripts. A fold change (FC) > 2 and *p *< 0.05 were considered to indicate significant differences.

### CircRNA expression by qRT-PCR

qRT-PCR was performed to validate microarray findings. Reverse transcription of 1 μg total RNA was carried out with PrimeScriptTM RT reagent Kit and gDNA Eraser Kit (Yingjun Biotechnology). Then, qRT-PCR was performed with SYBR Premix Ex Taq TM (TaKaRa) as instructed by the manufacturer. Transcript levels of circRNAs were evaluated, with Glyceraldehyde 3-phosphate dehydrogenase (GAPDH) as a reference gene. The primer sequences were shown in Table 1.

**Table 1:** Primers were shown for qPCR

Gene	Primer	Annealing temperature (°C)	Product length (bp)
GAPDH (human)	F:5′GGGAAACTGTGGCGTGAT3′ R:5′GAGTGGGTGTCGCTGTTGA3′	60	299
hsa_circ_058230	F:5′TGGATGGGGAGCCCTACAAG3′ R:5′CCAGGTGCGGGTGTACAGG3′	60	94
hsa_circ_0000257	F:5′GGAGCAGACCAAGGCAGCG3′ R:5′CGTCAAAGATCACGACTGTCCC3′	60	120
hsa_circ_0061170	F:5′CCAGAAGCCAAAGATAACACC3′ R:5′ATTTGCCTGTAACTTTCGCTC3′	60	155
hsa_circ_0065217	F:5′CCATGCCAATATGTGGGTGC3′ R:5′GCCAGGAGGTTCTTGTGCC 3′	60	89
hsa_circ_0087354	F:5′CTGGAGTAGGAGTTTGGTGGTA3′ R:5′CTTCACCAGAGGATGTATTGCT3′	60	64
hsa_circ_0001949	F:5′GTGCTGATCTTCTGACATTCAGGT3′ R:5′CTGGAAGCTCAGGATTATCTGGA3′	60	154

### Functional enrichment analysis and circRNAs/miRNAs associations

The circRNAs and miRNAs showing significant associations were analyzed. Possible response elements of miRNAs were searched in circRNAs and miRNAs sequences. Next, miRNA binding site prediction was searched with the miRcode map (http://www.microde.org/). The circRNAs/microRNAs interaction was searched with Arraystar’s home-made miRNA target prediction software based on TargetScan & MiRanda, and the differentially expressed circRNAs within all the comparisons were annotated in detail with the circRNAs/miRNAs interaction information. GO and KEGG Pathway analysis was carried out with standard techniques. GO enrichment analysis was based on three aspects: biological process (BP), cellular component (CC), and molecular function (MF) and GO analysis was carried out to assess the functional roles of the top 10 significant enriched target genes. KEGG pathway enrichment revealed the signaling networks of differentially circRNAs associated with oxidative stress in hDPSCs.

### SIRT1 gene expression by qRT-PCR

qRT-PCR was used to confirm the SIRT1 expression. Total RNA was extracted from cells using RNAiso Plus (9108Q, TaKaRa) and reversely transcripted into cDNA using PrimeScriptTM RT reagent Kit with gDNA Eraser (RR047A, TakaRa) following the manufacturer’s protocol. The relative gene expression was determined using Thermal cycler Dice Real Time System (TP800, TaKaRa) by SYBR Premix Ex TaqTM II (Tli RNaseH Plus, RR820A, TaKaRa). The Transcript levels of SIRT1 were evaluated with β-actin serving as the internal control standard. The primer sequences were as follows: F: TGTGGTAGAGCTTGCATTGATCTT, R: GGCCTGTTGCTCTCCTCATT. Data were shown as fold change (2^−∆∆Ct^) and analyzed initially using GraphPad Prism 7 software. Triplicates were performed for each sample in three independent experiments.

### In-Cell Western analysis for SIRT1

Following treatment, the cells were washed in PBS, followed by fixation in 10% neutral buffered formalin (NBF, Cellpath) for 20 minutes. Cells were permeabilized by washing five times in 0.1% Triton™ X-100 in PBS for 5 minutes per wash. Non-specific binding was blocked using the Odyssey® blocking buffer (Li-Cor Biosciences) for 1.5 h at room temperature. The samples were incubated with anti-SIRT1 (1:600) antibodies (Abcam) in Odyssey® buffer at 4 °C overnight with gentle shaking. Samples were washed extensively in PBS containing 0.1% Tween20 five times for 5 minutes per wash. Cells were incubated with the IRDye® 800CW secondary antibody (1:800) with the CellTag™ 700 stain (1:500; Li-Cor Biosciences) in the Odyssey^®^ blocking buffer for 1 hour at room temperature with gentle shaking. Samples were washed in PBS containing Tween20 five times for 5 min per wash. After the final wash, all liquid was removed and the plate was scanned on the Odyssey^®^ SA Imaging System (Li-Cor Biosciences) using both 700 and 800 nm detection channels at a 200 nm resolution, medium quality with a focus offset of 3.0 mm. Quantitative In-Cell Western (ICW) analysis was performed using Image Studio (Li-Cor Biosciences: version 5).

### Statistical analysis

The statistical significance of microarray data was analyzed in terms of fold change using the Student’s *t* test, and FDR was calculated to correct the *p*-value. FC > 2 and *p *< 0.05 were used to screen the differentially expressed circRNAs. For the gene expression and activity analysis, GraphPad Prism 7 software was applied. Student’s t-test was applied for comparison of two groups and differences with *p *< 0.05 were considered statistically significant.

## Results

### The growth of hDPSCs

**Fig. 1:**
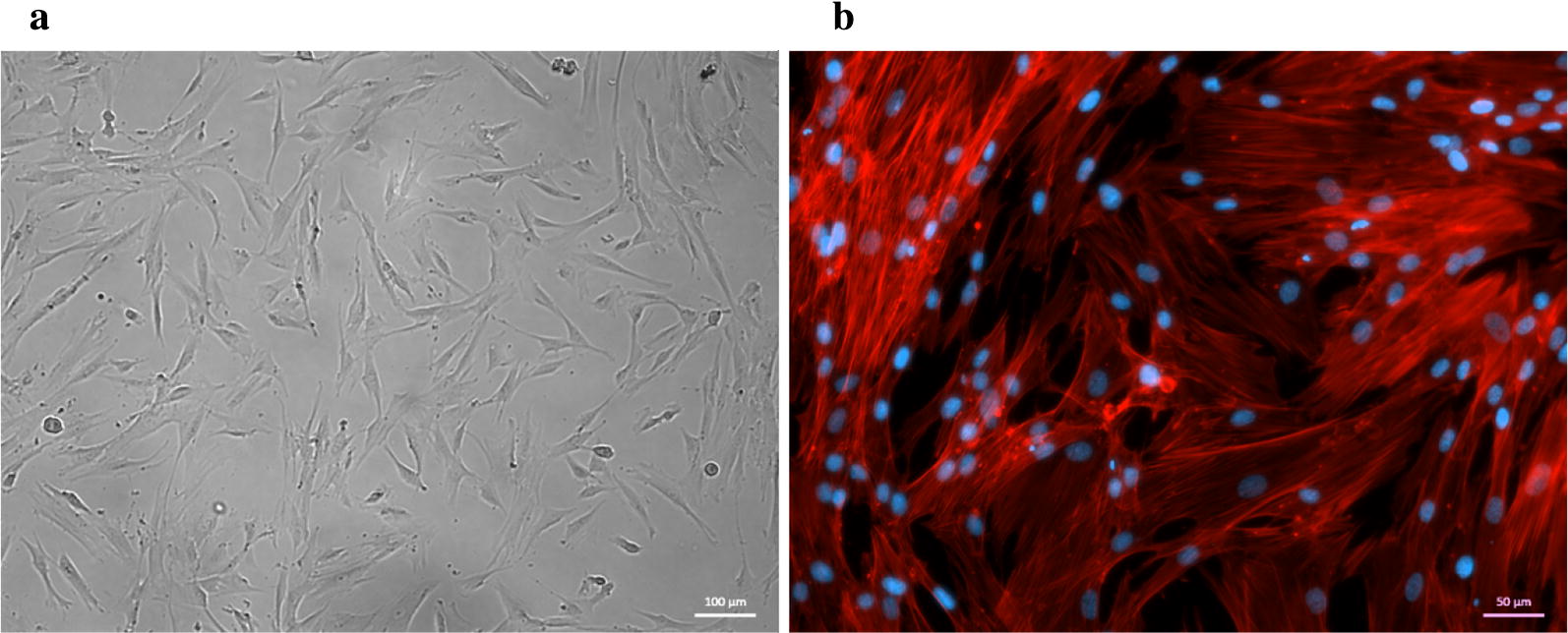
Morphology and F-actin staining of hDPSCs. **a** Brightfield of hDPSCs, **b** F-actin (red) & DAPI (blue) of hDPSCs

After 1 week of primary cell culture, the morphology of the extracted hDPSCs was fibroblast-like and polygon (Fig. 1a). The hDPSCs were stained for F-actin and nuclei by red and blue fluorescence (Fig. 1b). Cytoskeletal fibers are parallel and evenly distributed, arranged in order.

### Functional evaluation of the oxidative stress model of hDPSCs

**Fig. 2:**
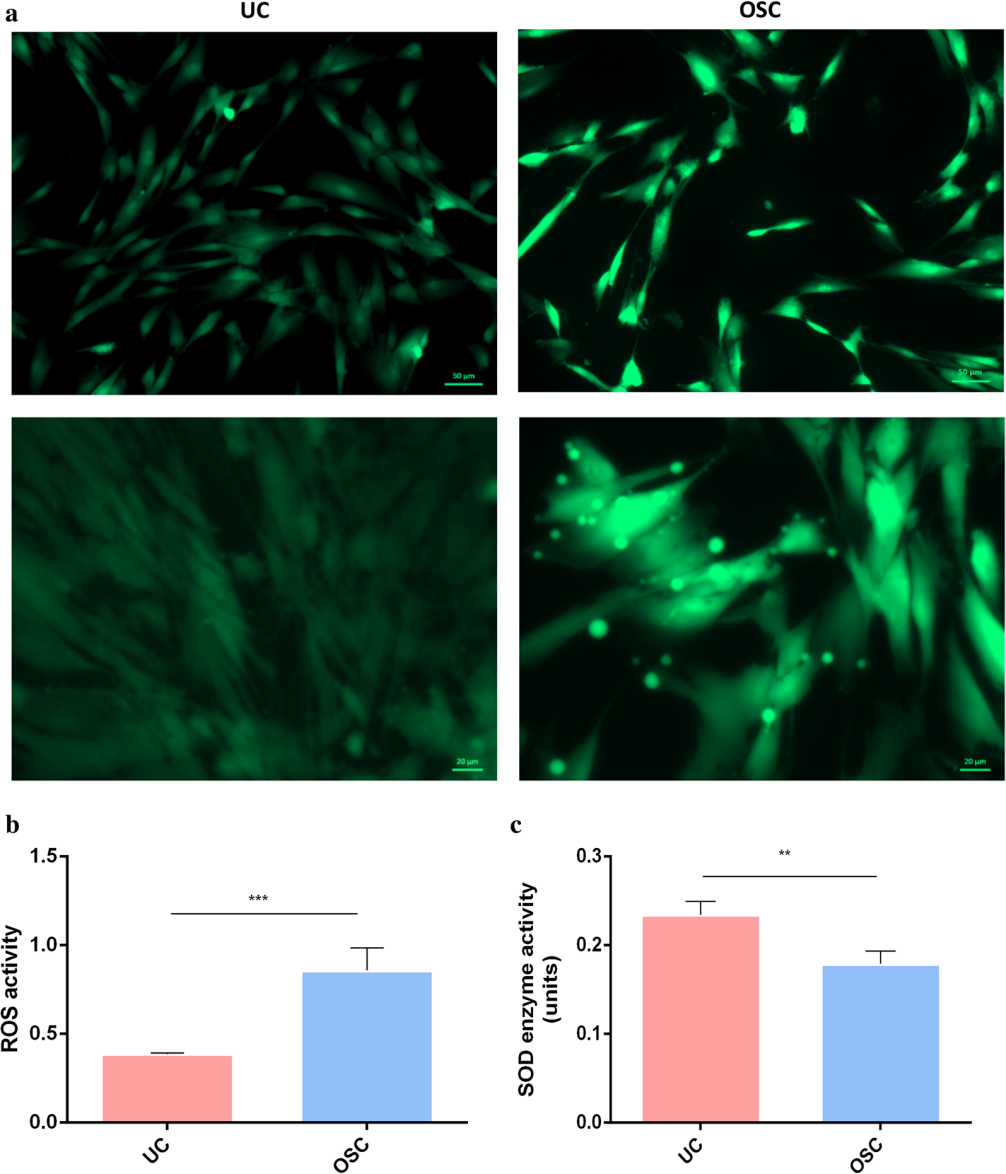
Fluorescence staining of ROS (Green) and ROS activity of NC and OSC. **a** ROS staining of hDPSCs treated with/without H_2_O_2_ (OSC/UC) for 24 h. **b** ROS activity and **c** SOD activity of UC and OSC. *p < 0.05, **p < 0.01, ***p < 0.001

After hDPSCs were treated by 0.2 mM H_2_O_2_ for 24 h, ROS levels within the cells were detected by fluorescent staining of ROS and activity analysis (Fig. 2a–c). From Fig. 2, positive ROS staining was located within both the nucleus and cytoplasm of the H_2_O_2_ treated cells. In the UC group, ROS was weekly detected compared with those of H_2_O_2_ treated cells. ROS activity analysis also provided evidence that ROS activity was increased in the H_2_O_2_ treated cells. Both the fluorescence and activity analysis results confirmed that 0.2 mM H_2_O_2_ treatment for 24 h induced oxidative stress in cells. In the subsequent experiment, hDPSCs which were treated by 0.2 mM H_2_O_2_ for 24 h was used as the model of OSCs.

### Identification and quantification of human circRNAs

**Fig. 3:**
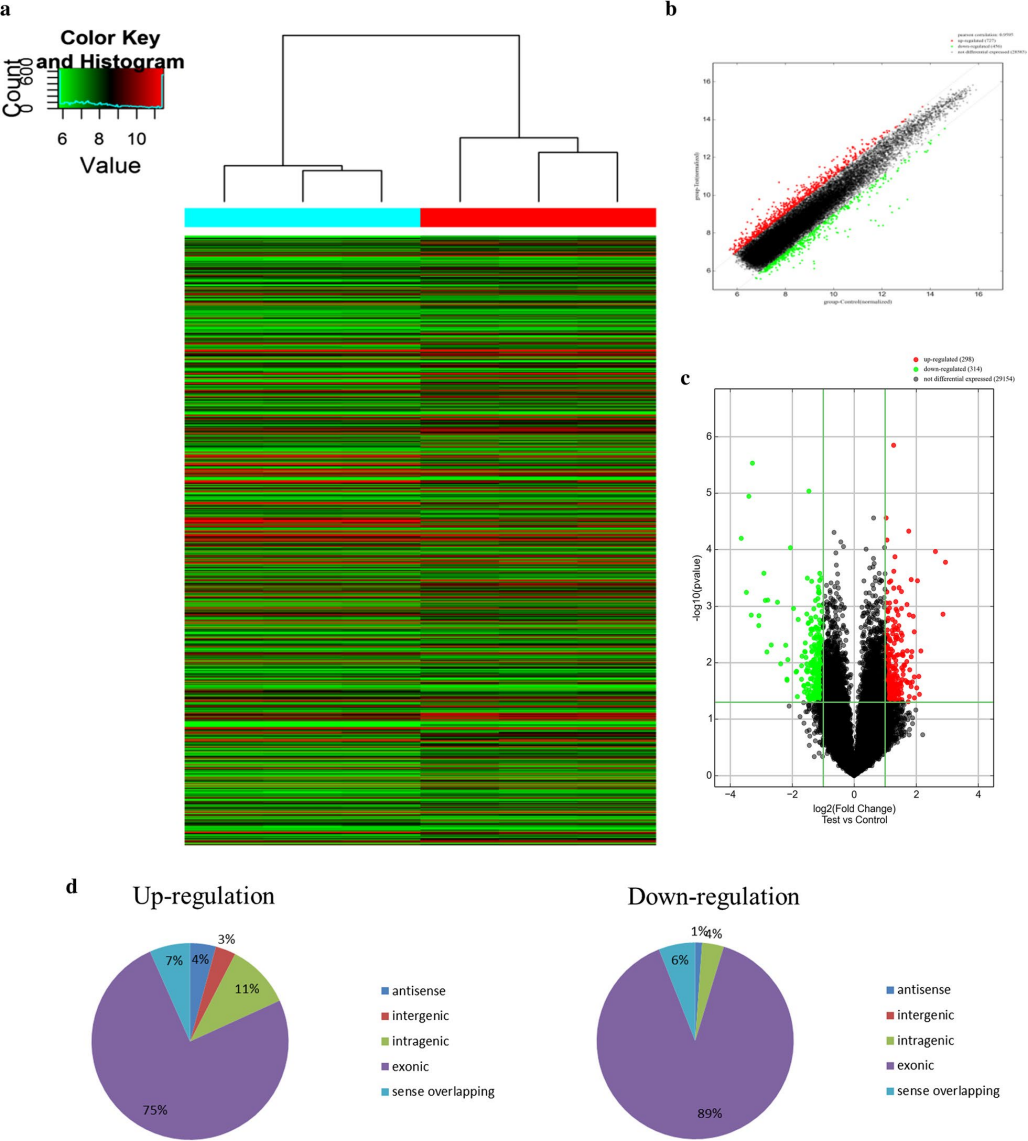
Differential expression of circRNAs in UC and OSC. **a** Hierarchical clustering analysis of circRNAs that were differentially expressed between OSC and UC samples; each group contains three individuals (greater than two-fold difference in expression; p < 0.05). Expression values are represented in different colors, indicating expression levels above and below the median expression level across all samples. **b** The scatter plot is a visualization method used for assessing the variation in circRNA expression between OSC and UC. The values corresponding to the X- and Y-axes in the scatter plot are the normalized signal values of the samples (log_2_ scaled). The green lines indicate fold changes. The circRNAs above the top green line and below the green bottom line indicate more than twofold changes between OSC and UC samples. **c** Volcano plots were constructed using fold-change values and p-values. The vertical lines correspond to twofold up- and down-regulation between OSC and UC, and the horizontal line represents a p-value. The red point in the plot represents the differentially expressed circRNAs with statistical significance (OSC: hDPSCs treated by 0.2 mM H_2_O_2_ for 24 h. UC: untreated hDPSCs). **d** Classification of dysregulated circRNAs

**Fig. 4:**
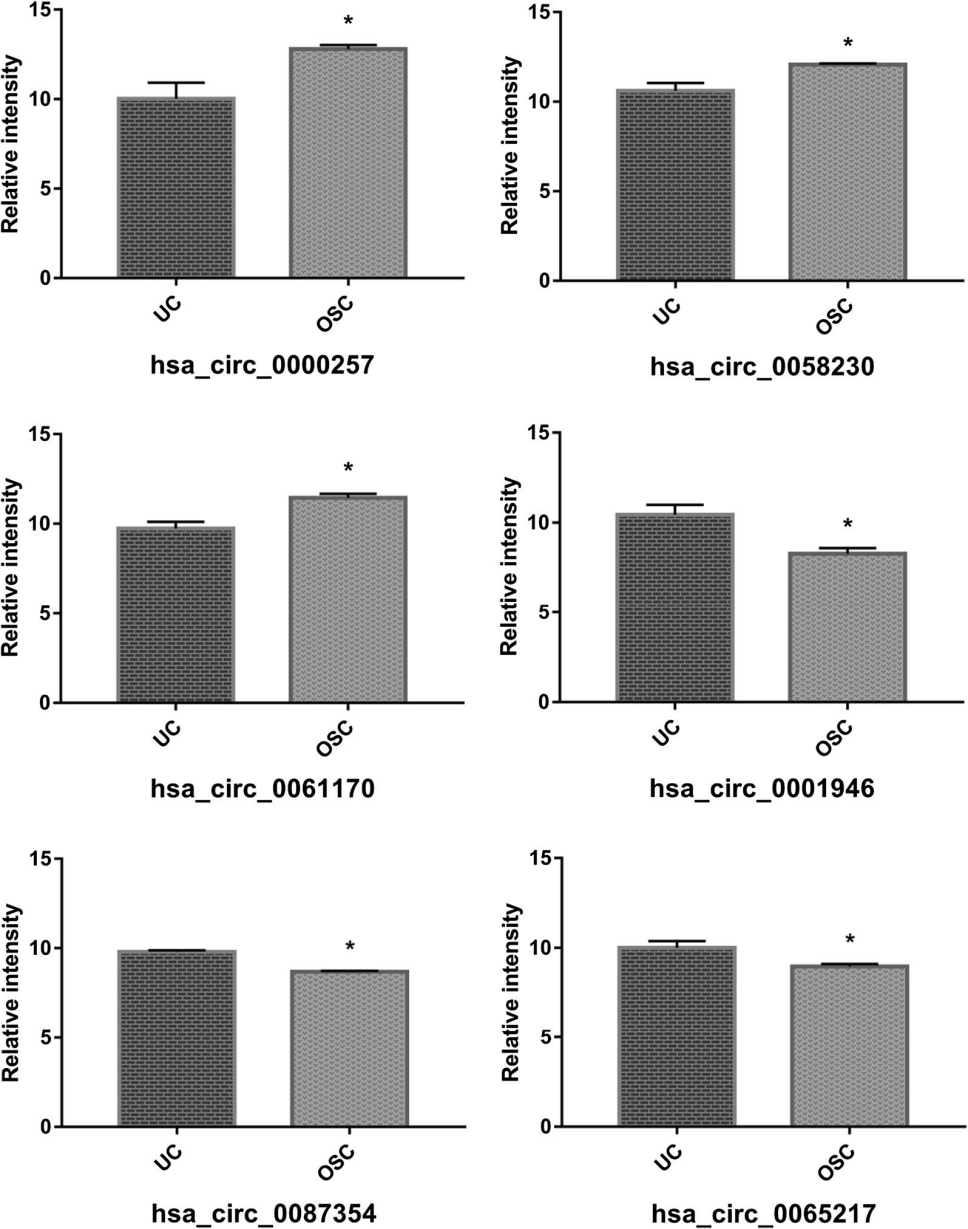
Relative intensity of the top three upregulated circRNAs (hsa_circ_058230, hsa_circ_0061170, and hsa_circ_0000257) and downregulated circRNAs (hsa_circ_0065217, hsa_circ_0087354, and hsa_circ_0001946) *p < 0.05

Hierarchical clustering revealed multiple circRNAs expression in the UC and OSC group (Fig. 3a). The scatter ad volcano plots showed the variation of circRNA expression between the UC and OSC group (Fig. 3b, c). A total of 863 circRNAs were identified in OSC and UC. 330 circRNAs were upregulated, while 533 were downregulation (fold change cutoff 2; *p *< 0.05) in OSC compared with UC. Among the circRNAs within OSCs, hsa_circ_058230, hsa_circ_0061170, and hsa_circ_0000257 were upregulated by 6.830, 2.77, and 3.26-fold, respectively. While, hsa_circ_0065217, hsa_circ_0087354, and hsa_circ_0001946 were downregulated in OSC by 2.04, 2.16, and 4.48-fold, respectively. The six circRNAs expression variation was most significant between OSC and UC among the 330 upregulated circRNAs, and 533 downregulated circRNAs, which were shown in Table 2 and Fig. 4. Therefore, we will focus the six circRNAs for the subsequent experiments.

**Table 2:** The list of the top three upregulated and downregulated circRNAs

Alias	circRNA	FC	p	FDR	Circ Start	Circ End	circRNA type
hsa_circ_0000257	hsa_circRNA_100674	6.832904	0.006702	0.062766	103916775	103917971	Exonic
hsa_circ_0058230	hsa_circRNA_058230	2.772335	0.004413	0.052862	219204505	219206867	Exonic
hsa_circ_0061170	hsa_circRNA_061170	3.255504	0.002475	0.040873	62303908	62305446	Exonic
hsa_circ_0001946	hsa_circRNA_105055	4.4751	0.00395	0.049809	139865339	139866824	Antisense
hsa_circ_0087354	hsa_circRNA_104803	2.16559	5.05E−05	0.015259	86292641	86292876	Exonic
hsa_circ_0065217	hsa_circRNA_103349	2.043447	0.011242	0.079325	47468646	47470160	Exonic

### qRT-PCR of representative circRNA

**Fig. 5:**
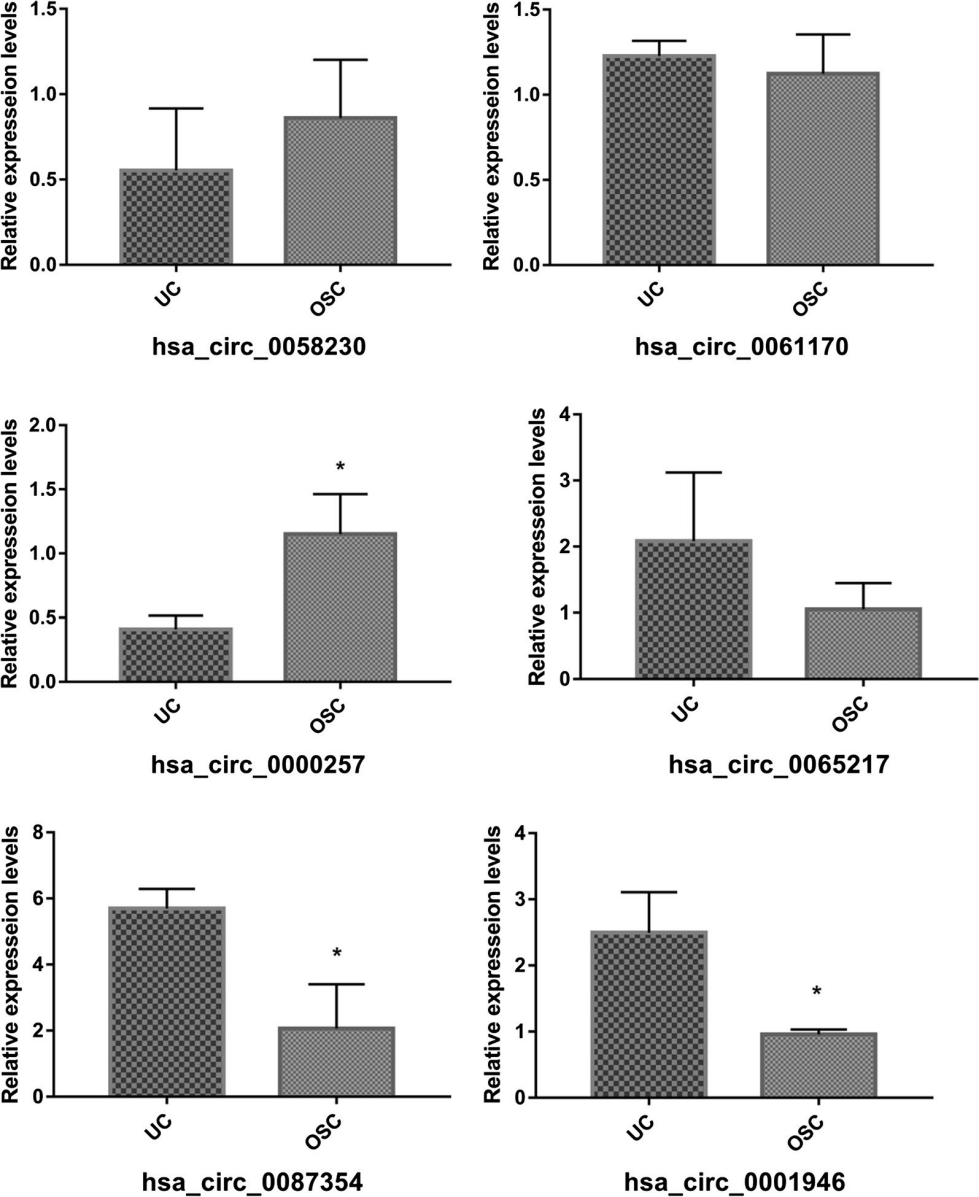
qRT-PCR verification of differentially expressed circRNAs (hsa_circ_058230, hsa_circ_0061170, hsa_circ_0000257, hsa_circ_0065217, hsa_circ_0087354, and hsa_circ_0001946), which was consistent with the sequencing results. *p < 0.05

To validate the circRNA microarray results, the six dysregulated circRNAs were selected as the typical representative, which exhibited significant changes in expression among the differentially expressed circRNAs. qRT-PCR experiments were performed to analyze the gene expression changes between the OSC and UC group. Hsa_circ_058230, hsa_circ_061170, and hsa_circ_0000257 were selected as representatives of up-regulated circRNAs, while hsa_circ_0065217, hsa_circ_0087354, and hsa_circ_0001946 were assessed as down-regulated circRNAs. The circRNA’s expression of hsa_circ_0000257 was significantly up-regulated (2.84-fold) in OSC compared to the UC group (*p *= 0.018) (Fig. 5), and the microarray analysis showed a similar trend with OSC, 6.83-fold higher than UC. For hsa_circ_0058230 (*p *= 0.341) and hsa_circ_0061170 (*p *= 0.505), there were no significant differences between OSC and UC by qRT-PCR. For the down-regulated circRNAs, qRT-qPCR showed that hsa_circ_0087354 (*p *= 0.013) and hsa_circ_0001946 (*p *= 0.013) were downregulated by 2.94 and 2.70-fold in OSC group compared with UC, respectively. These findings corroborated the microarray data. There was no significance observed between the OSC and UC by qRT-PCR for hsa_circ_0065217 (*p *= 0.183).

### Prediction of the circRNA/microRNA interaction

**Fig. 6:**
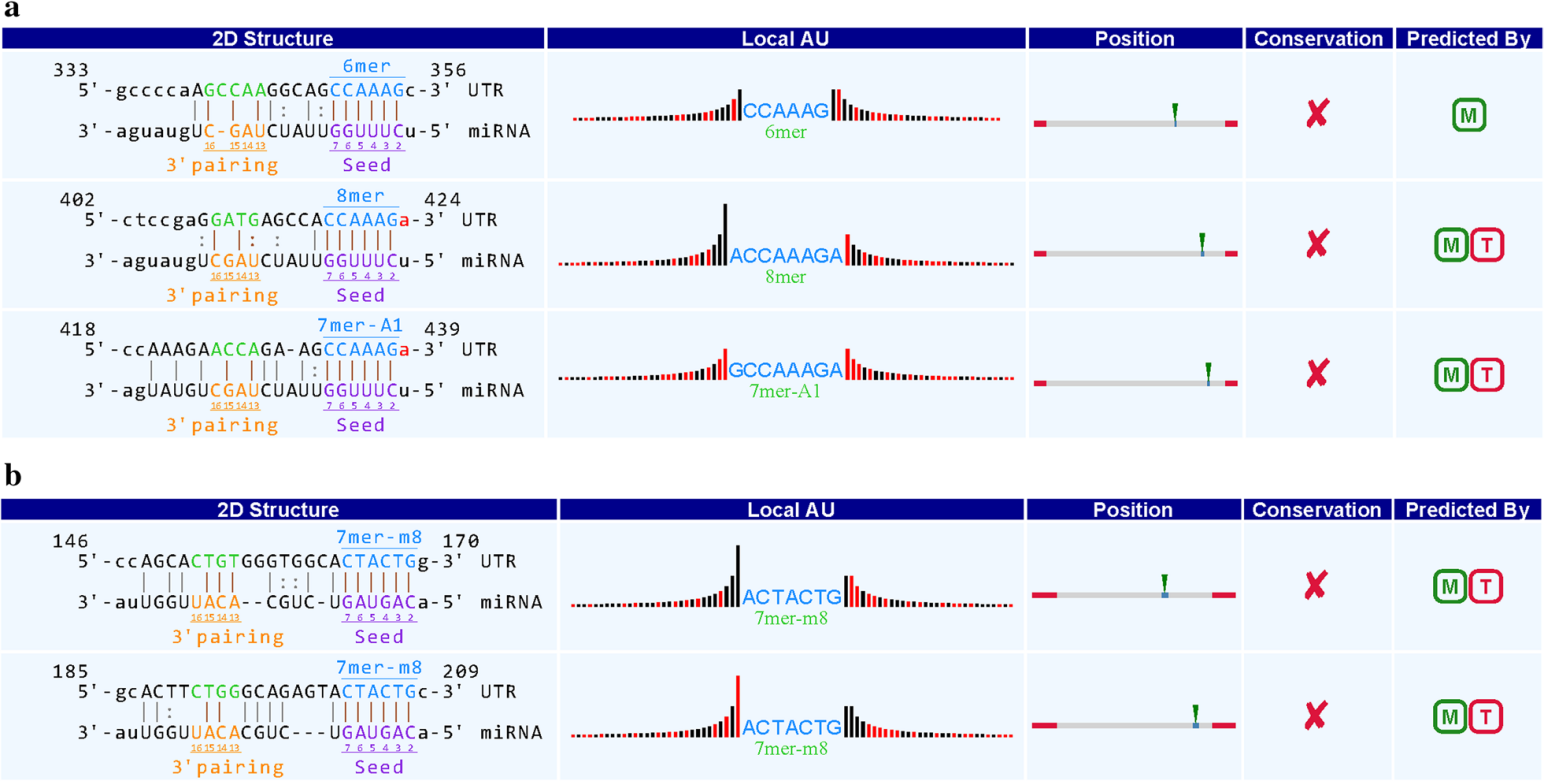

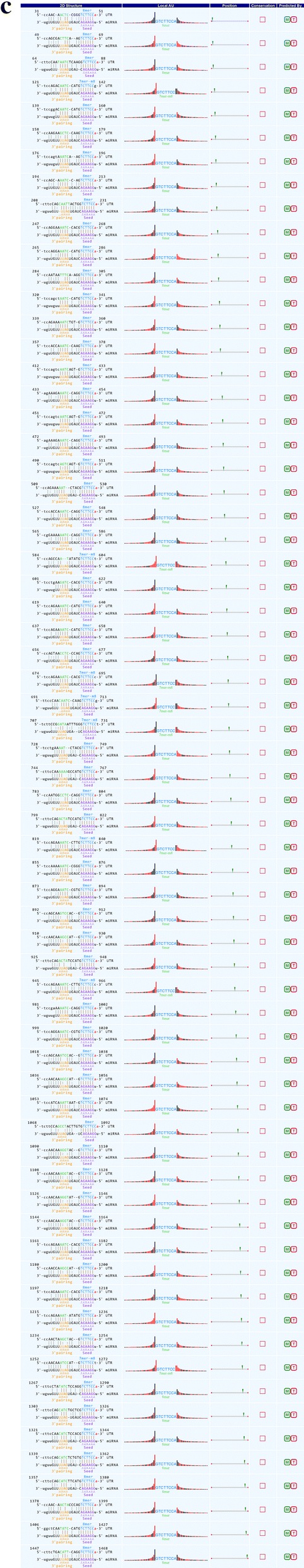
A snippet of the detailed annotation for circRNA/miRNA interaction [**a** hsa_circ_0000257 vs hsa-miR-9-5p; **b** hsa_circ_0087354 vs hsa-miR-199a-3p and **c** hsa_circ_0001946 vs hsa-miR-7-5p)]

To find the potential miRNA target, three differentially expressed circRNAs (hsa_circ_0000257, hsa_circ_0087354, and hsa_circ_0001946), which were confirmed by qRT-PCR were selected and the circRNA/miRNA interaction was predicted with Arraystar’s home-made miRNA target prediction software based on TargetScan & miRanda. The potential miRNA targets of hsa_circ_0000257 include hsa-miR-9-5p, hsa-miR-647, hsa-miR-653-3p, hsa-miR-212-5p and hsa-miR-27a-5p. For hsa_circ_0087354, the potential miRNA targets include hsa-miR-199a-3p, hsa-miR-199b-3p, hsa-miR-449a, hsa-miR-449b-5p and hsa-miR-630. Finally, hsa_circ_0001946 could interact with microRNAs, including hsa-miR-7-5p, hsa-miR-3529-5p, hsa-miR-8056, hsa-miR-1246 and hsa-miR-139-3p. These representative interactions of miRNAs and circRNAs were represented in Fig. 6.

### Functional analysis of differentially expressed circRNAs

**Fig. 7:**
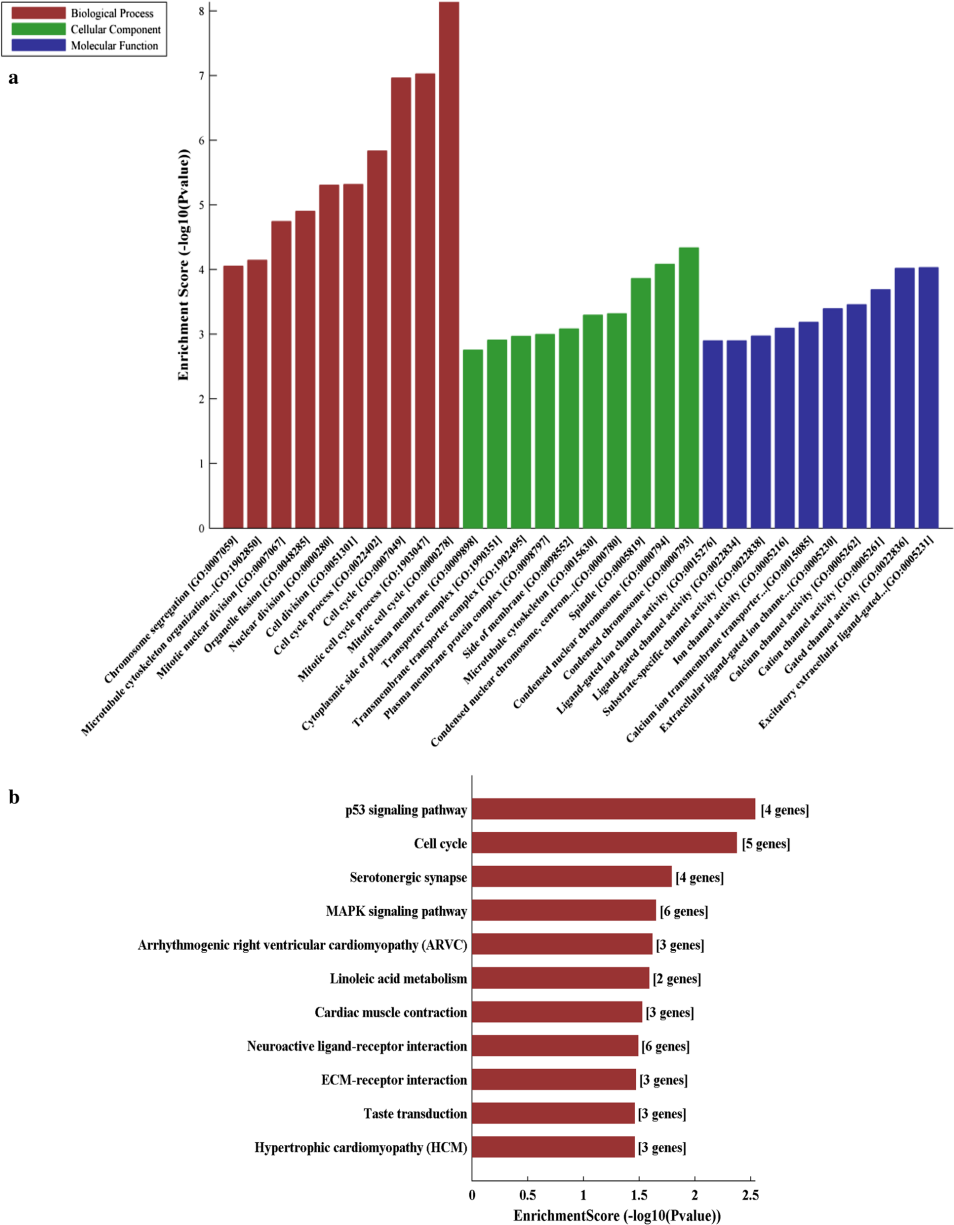
GO and KEGG enrichment terms of differentially expressed circRNAs transcript genes. **a** Top ten enrichment score covering domains of biological processes, cellular components, and molecular function. **b** KEGG pathway enrichment analysis of with top ten enrichment score

The differentially expressed circRNA genes were analyzed by GO (Fig. 7a) and KEGG (Fig. 7b) enrichment. Based on the results, these differentially expressed circRNAs may be associated with GO functional annotation of biological processes (*e.g*., mitotic cell cycle, cell cycle, mitotic cell cycle process), cellular components (*e.g.*, condensed chromosome, condensed nuclear chromosome, spindle), molecular function (*e.g.*, excitatory extracellular ligand-gated, gated channel activity, cation channel activity). According to KEGG analysis, the host genes of these differentially expressed circRNAs are associated with the p53 signaling pathway, cell cycle, serotonergic synapse, MAPK signaling pathway in oxidatively stressed hDPSCs. In particular, the p53 signaling pathway plays an important role in a variety of oxidative stress.

### SIRT1 gene and protein expression

**Fig. 8:**
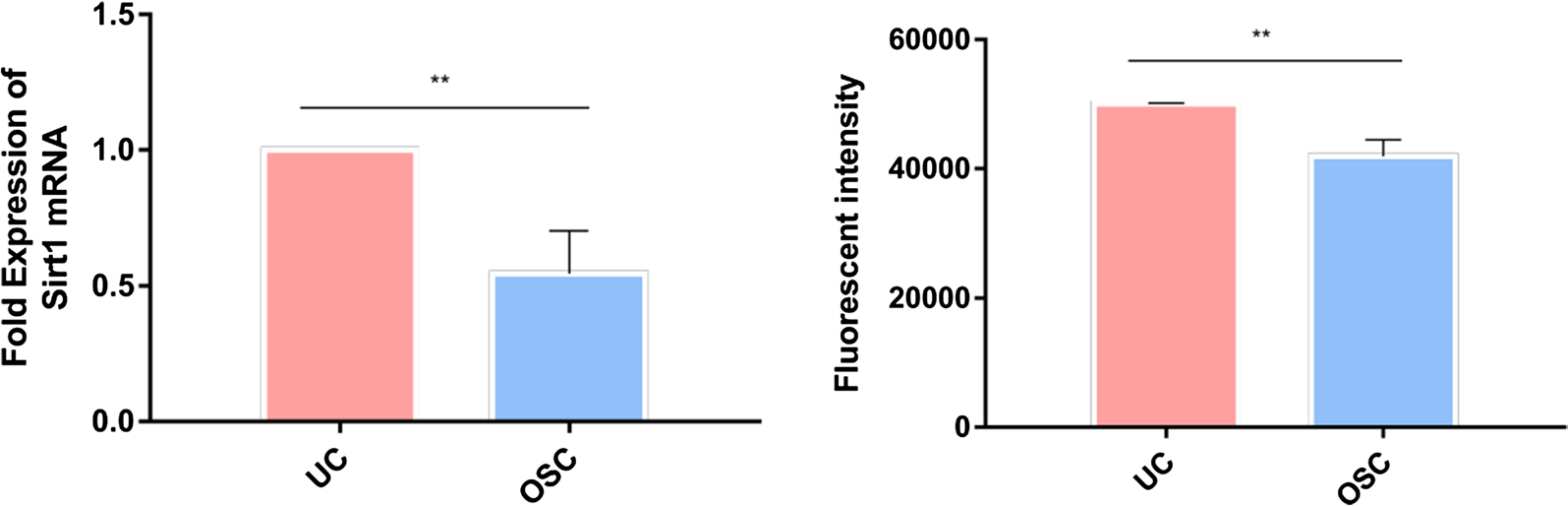
The effects of H_2_O_2_ on hDPSCs SIRT1 mRNA and proteins expression. Data expressed as mean ± SD (n = 3). **p < 0.01

The mRNA levels of SIRT1 in UC and OSC group were shown in Fig. 8. The SIRT1 mRNA expression exhibited a 0.055-fold reduction in the OSC group compared to that in the UC (p < 0.01). Similar findings were observed at the protein level, where ICW analysis confirmed a 1.19-fold reduction in SIRT1 protein expression following H_2_O_2_ exposure.

## Discussion

hDPSCs have garnered increasing attention as a potential MSCs source for regenerative medicine due to several advantages including increased proliferation rate and ease of procurement [6]. It has been shown that oxidative stress impairs the capability of MSCs to proliferate and differentiate into multiple lineages [7, 8]. Numerous studies have shown the involvement of circRNAs in the process of oxidative stress [9, 10]. Kristensen et al. found that circRNAs show elevated expression levels during the differentiation of human epidermal stem cells [11]. Liu et al. reported that cZNF609 regulates MEF2A and is likely involved in oxidative stress [12]. Indeed, as miRNA sponges [13, 14], circRNAs control the expression of parent genes to regulate oxidative stress in endothelial cells [12] and cancer cells [15]. However, the functions of circRNAs in oxidative stress remain undefined in hDPSCs. Microarray analysis showed that 330 and 533 circRNAs were markedly upregulated and downregulated by oxidative stress in hDPSCs compared with untreated cells, respectively. Of these, hsa_circ_058230, hsa_circRNA_0061170, and hsa_circ_0000257 were the most distinctly upregulated during oxidative stress, while hsa_circ_0065217, hsa_circ_0087354, and hsa_circ_0001946 exhibited the most significant degree of downregulation. These findings were validated by qRT-PCR and suggested that hsa_circ_0000257 was upregulated, and hsa_circ_0087354 and hsa_circ_0001946 were downregulated involved in the post-transcriptional regulation of oxidative stress in hDPSCs.

Recent evidence has demonstrated that circRNAs play a crucial role in fine-tuning the level of miRNA mediated regulation of gene expression by sequestering the miRNAs [16, 17]. Their interaction with diseases associated miRNAs indicates that circRNAs are essential for disease regulation. CircRNAs are considered to adversely regulate miRNAs, substantially contributing to the competing endogenous RNA (ceRNA) network [18, 19]. Research has demonstrated that ciRS-7, a circular miR-7 inhibitor, comprises > 60 popular miR-7 binding sites [14], a number substantially higher than reported for any known linear sponge. As shown above, hsa_cir_0000257 regulated 128 microRNAs, while the downregulated circRNAs hsa_circ_0087354 and hsa_cir_0001946 regulated 58 and 123 microRNAs, respectively.

It is therefore essential to further assess the newly identified dysregulated circRNAs, and unveil their biological roles in oxidative stress. CircRNAs regulate the neighboring and overlapping coding genes, with effects embodied in the associated mRNA-producing genes. It has been shown that miRNAs could regulate the expression level of mRNAs on stromal cells to decrease oxidative stress damage, while the function of the most potential miRNAs on hDPSCs are far from clear. Here, GO and KEGG pathway analysis was performed to assess the functions of associated miRNAs. GO annotation showed that the identified target genes regulated critical biological processes, indicating that modulating genes is critical in oxidative stress. P53 signaling pathway, cell cycle, serotonergic synapse, MAPK signaling pathway were involved in regulating oxidative stress in hDPSCs.

The induced pathways highlighted by KEGG analysis included the p53 pathway, which might be a key mediator of oxidative stress in hDPSCs. It was demonstrated that p53 signaling corresponding to repressed circRNAs plays essential roles in oxidative stress [20]. Various forms of oxidative stress lead to post-translational modifications of p53, allowing it to regulate genes to cause either beneficial outcomes, such as the upregulation of mitochondrial biogenesis, or more dysfunctional consequences such as cellular senescence and apoptosis [21]. Several studies in different cells systems have confirmed that p53 is the downstream gene of SIRT1. Liu et al. demonstrated that SIRT1 could bind to p53, reduce its acetylation level by co-immunoprecipitation assay, and treatment with outline-3A reversed the effect of SIRT1 on the level of p53 in adipose-derived stromal cells [22]. Moreover, Shi et al. also confirmed that activation of SIRT1 using its agonist resveratrol ameliorated cellular apoptosis via deacetylating p53 [23]. The findings of this present study indicate that the p53 signaling pathway might play a critical role in regulating oxidative stress of hDPSCs, which is consistent with previous studies [7, 24, 25].

Additionally, studies have reported in response to oxidative stress, p38 MAPK can rapidly phosphorylate and activate MAP kinase-activated protein kinases [26]. It is well known that oxidative stress halts cell cycle progression and ultimately results in initiating cell death [26]. Du et al. reported that circ-FOXO3 halts cell cycle progression via interacting with P21 and CDK2 proteins [27]. Therefore, it is likely that circRNAs play a similar role in oxidative stress.

Based on the findings of this study, we hypothesize that hsa_cir_0000257 specifically binds and inhibits several miRNAs such as hsa-miR-647, hsa-miR-653-3p, hsa-miR-9-5, and hsa-miR-27a-5p. Among them, SIRT-1 30UTR and miR-9-5p (50eUCUUUGGU-30) recognized by the TargetScan algorithm are highly conserved complementary sequences. The SIRT1 gene expression was downregulated in OSC compared with UC, which was validated by qRT-PCR. Moreover, D’ Adamo S’s group confirmed that SIRT1 is a direct target gene of miR-9 in human primary and C-28/12 chondrocytes by luciferase reporter assay, while qRT-PCR and western blot analysis confirmed miR-9 targeting SIRT1 regulates oxidative stress damage in human primary and C-28/12 chondrocytes [28]. Saunder et al. also demonstrated that miR-9 targets SIRT1 to regulate the expression of embryonic stem cell differentiation in mouse embryonic stem cells [29]. Therefore, the hsa_circ_0000257/hsa-miR-9-5p/SIRT1/P53 regulatory axis is likely a novel molecular pathway regulating oxidative stress in hDPSCs. Although most circRNAs are not well-understood, the potential targets of the altered miRNAs were assessed.

## Conclusions

Together, specific circRNAs are involved in oxidative stress of hDPSCs. Further, qRT-PCR analysis unveiled hsa_circ_0087354 and hsa_circ_0001946 were down-regulated circRNAs, as well as hsa_cir_0000257 was up-regulated circRNAs for the OSC comparing to UC. Pathway analysis revealed that p53 signaling might participate in oxidative stress. The hsa_circ_0000257/hsa-miR-9-5p/SIRT1/P53 regulatory axis is likely a novel molecular pathway regulating oxidative stress in hDPSCs. The current findings provide a basis for assessing circRNA functions in oxidatively stressed hDPSCs.

## Data Availability

The datasets used or analyzed during the current study are available from the corresponding author on reasonable request.

## Abbreviations

hDPSCs: Human Dental Pulp Stromal Cells
circRNAs: circular RNAs
qRT-PCR: quantitative real-time PCR
GO: gene ontology
KEGG: Kyoto Encyclopedia Genes and Genomes
miRNAs: microRNAs
α-MEM: alpha-modified Minimum Essential Medium
FBS: fetal bovine serum
UC: untreated cells
Ds-cDNA: double-stranded cDNA
FC: fold change
GAPDH: glyceraldehyde 3-phosphate dehydrogenase
OSC: oxidative stressed cells
ceRNA: competing endogenous RNA
